# Supplementary material for: NADH-GOGAT Overexpression Does Not Improve Maize (Zea mays L.) Performance Even When Pyramiding with NAD-IDH, GDH and GS
Source: Plants (Basel). 2020 Jan 21;9(2):130. doi: 10.3390/plants9020130 (PMC7076717; doi:10.3390/plants9020130)
Supplement: Supplementary file 1 [file plants-09-00130-s001.zip › Supplemental data/Supplemental data proofs.docx]

| **Line** | **Plasmid** | **Construct characteristics** |
| --- | --- | --- |
| T02291 | pBIOS02120 | Actin_actin - TaNADH-GOGAT - terAtSac66 |
| T02289 | pBIOS02750 | Actin_actin - TaNADH-GOGAT - terAtSac66 / CsVMV_actin - SbNAD_IDH - terZmHSP |
| T02308 | pBIOS02789 | Actin_actin - TaNADH-GOGAT - terAtSac66 / CsVMV_actin - SbNAD_IDH - terZmHSP / Actin_actin - ZmGDH1 - terZmHSP |
| T02312 | pBIOS02792 | Actin_actin - TaNADH-GOGAT - terAtSac66 / CsVMV_actin - SbNAD_IDH - terZmHSP / Actin_actin - ZmGDH1 - terZmHSP / CsVMV_actin - ZmGS1.3 - terZmGS1.3 - terAtNOS / ZmrbcS - ZmGS1.3 - terZmGS1.3 - terAtNOS |

**Table S1.** Characteristics of the different constructs used for maize transformation.

- T02291 contains the *Triticum aestivum* (Ta) NADH-dependent glutamate synthase (NADH)-GOGAT cDNA (TraesCS3B01G299800) under the control of the rice Actin1 promoter [1] followed by its first intron and the Arabidopsis (At) endopolygalacturonase1 (Sac66) terminator.
- T02289 contains the *Triticum aestivum* NADH-dependent glutamate synthase (NADH)-GOGAT cDNA under the control of the rice Actin1 promoter followed by its first intron and the Arabidopsis Sac66 terminator + the sorghum (Sb) NAD-dependent isocitrate dehydrogenase (NAD-IDH) cDNA (Sb04g024840) under the control of the Cassava Vein Mosaic Virus (CsVMV) promoter [2]. followed by the rice Actin1 first intron and the maize (Zm) Heat Shock Protein (HSP) terminator.
- T02308 contains the *Triticum aestivum* NADH-dependent glutamate synthase (NADH)-GOGAT cDNA under the control of the rice Actin1 promoter followed by its first intron and the Arabidopsis (Sac66) terminator + the sorghum (Sb) NAD-dependent isocitrate dehydrogenase (NAD-IDH) cDNA under the control of the CsVMV promoter followed by the rice Actin1 first intron and the maize HSP terminator + the maize NADH-dependent glutamate dehydrogenase 1 (GDH1) cDNA (GRMZM2G178415) under the control of the rice Actin1 promoter followed by its first intron and the maize HSP terminator.
- T02312 contains the *Triticum aestivum* NADH-dependent glutamate synthase (NADH)-GOGAT cDNA under the control of the rice Actin1 promoter followed by its first intron and the Arabidopsis Sac66 terminator + the sorghum NAD-dependent isocitrate dehydrogenase (NAD-IDH) cDNA under the control of the CsVMV promoter followed by the rice Actin1 first intron and the Arabidopsis Sac66 terminator + the maize NADH-dependent glutamate dehydrogenase 1 (GDH1) cDNA under the control of the rice Actin1 promoter followed by its first intron and the maize HSP terminator + two copies of the maize *Gln1.3* full length cDNA (D14577) + terminator [3], one flanked by the CsVMV promoter fused to the rice actin1 first intron and the Agrobacterium NOS terminator and the other one flanked by the promoter of the maize Rubisco small subunit (*RbcS*), [4] and the Agrobacterium NOS terminator.

**References**

1. Weckwerth, W.; Wendel, K.; Fienh, O. Process for the integrated extraction, identification and quantification of metabolites, proteins and RNA to reveal their co-regulation in biological networks. *Proteomics* **2004**, *4*, 78–83.
2. Verdaguer, B.; de Kochko, A.; Fux, C.I.; Beachy, R.N.; Fauquet, C. Functional organization of the cassava vein mosaic virus (CsVMV) promoter. *Plant Mol. Biol.* **1998**, *37*, 1055–1067.
3. Sakakibara, H.; Kawabata, S.; Hase, T.; Sugiyama, T. Differential effect of nitrate and light on the expression of glutamine synthetase and ferredoxin-dependent glutamate synthase in maize. *Plant Cell Physiol.* **1992**, *33*, 1193–1198.
4. Lebrun, M.; Waksman, G.; Fressinet, G. Nucleotide sequence of a gene encoding corn ribulose-1,5-bisphosphate carboxylase/oxygenase small subunit (rbcs). *Nucl. Acid Res.* **1987**, *15*, 4360.

| Target gene | Forward primer | Reverse primer |
| --- | --- | --- |
| GDH1 | TCTCCAAGAAGGGCGTGCT | CCACTCGAAGTACGACACCGT |
| NAD-ICDH | AGTTCCCCTCGTTCGCTGA | CCTTAGTCCTGTACTTGCCCTCC |
| NADH-GOGAT | CGAGGAGGAGGACGATGTGA | AACTTCGGGAGCAGCGTATC |
| GS1.3 | ACCACCATCATCTGGAAGCC | AGCAAACGGAACAGAAGGAACA |

**Table S2:** List of primers used for RT-qPCR experiments

**Figure S1.** Transcript abundance for NADH-GOGAT, NAD-IDH, NADH-GDH1 and GS1.3 in the leaves of transgenic plants overexpressing NADH-GOGAT (construct T02291) or stacked with the three other genes (constructs T02289, T02308 and T02312). The second numbers after each construct number represent the different transgenic events obtained following maize transformation. The untransformed wild type (WT line A188) was used as a control. mRNA quantification was performed using qRT-PCR with total RNA samples extracted from leaves at the vegetative (V) stage. Each value was normalized with the relative transcript abundance of the *EF1α* gene. Values correspond to that obtained using a pool of leaves harvested from four different plants for each transgenic event. The red asterisk above the bars corresponds to the transgenic events selected for metabolome analysis.


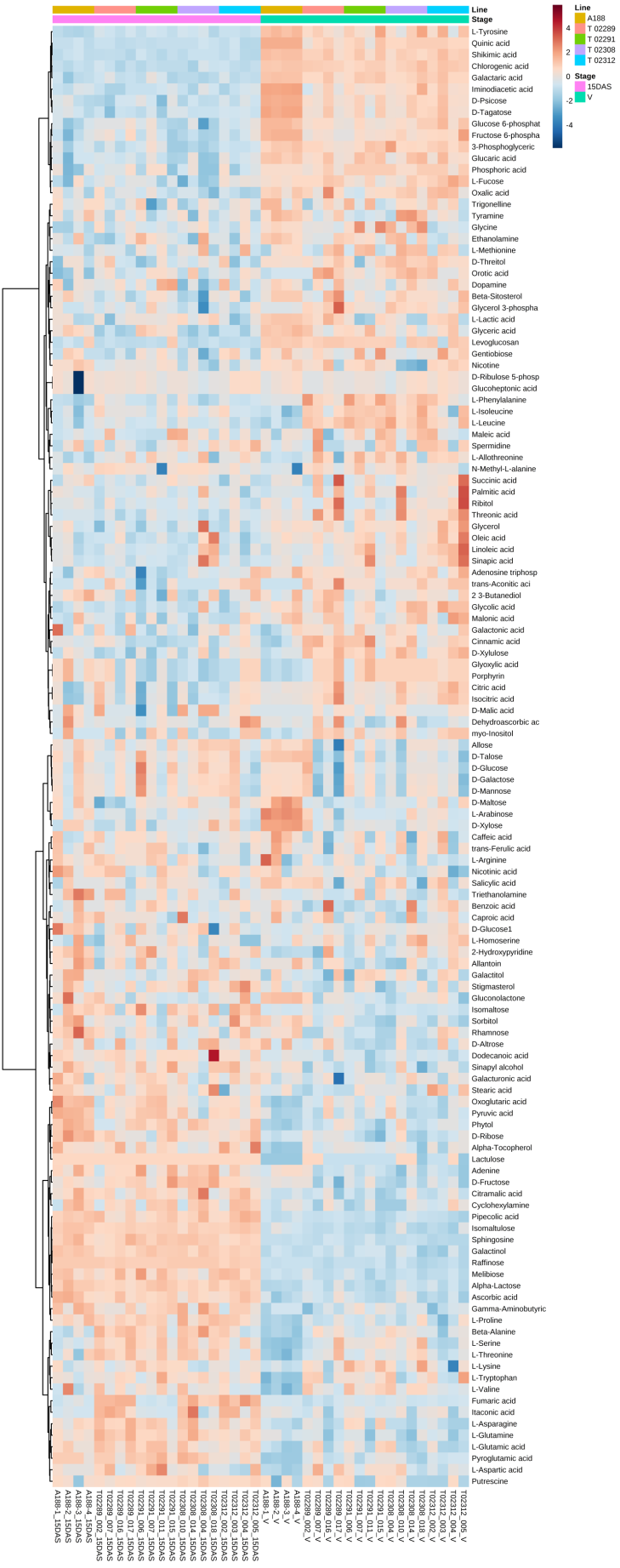


**Figure S2**. Hierarchical clustering analysis (HCA) of metabolites and at the V stage (purple horizontal bar) and 15DAS (green horizontal bar) in the WT (A188) plants and in the transgenic maize lines overexpressing NADH-GOGAT (construct T02291) and stacked with NAD-IDH (construct T02289), NAD-IDH + NADH-GDH1 (construct T02308) and NAD-IDH + NADH-GDH1 + GS1.3 (construct T02312). The intensity of the blue and brown colors corresponds to a lower and higher amount of metabolite (scale at the top of the figure).

**

**

**Figure S3.** Correlation matrix for the leaf metabolite content at the V stage. The heat map of the correlation matrix is based on the calculation of Pearson coefficients. Hierarchical clustering was performed using the Euclidean distances and Complete-linkage methods. The darkest red squares represent coefficients that are the closest to 1. The darkest blue squares represent coefficients that are the closest to -1. White squares represent coefficients that are close to 0 (see scale). The 6 group of co-regulated metabolites are separated by vertical white lines based on the hierarchical clustering.
